# Role of Endothelial Soluble Epoxide Hydrolase in Cerebrovascular Function and Ischemic Injury

**DOI:** 10.1371/journal.pone.0061244

**Published:** 2013-04-09

**Authors:** Wenri Zhang, Catherine M. Davis, Matthew L. Edin, Craig R. Lee, Darryl C. Zeldin, Nabil J. Alkayed

**Affiliations:** 1 Department of Anesthesiology and Perioperative Medicine, Oregon Health and Science University, Portland, Oregon, United States of America; 2 Division of Intramural Research, National Institute for Environmental Health Sciences (NIEHS), National Institutes of Health, Research Triangle Park, North Carolina, United States of America; 3 Division of Pharmacotherapy and Experimental Therapeutics, Eshelman School of Pharmacy, University of North Carolina, Chapel Hill, North Carolina, United States of America; Albany Medical College, United States of America

## Abstract

Soluble Epoxide Hydrolase (sEH) is a key enzyme in the metabolism and termination of action of epoxyeicosatrienoic acids, derivatives of arachidonic acid, which are protective against ischemic stroke. Mice lacking sEH globally are protected from injury following stroke; however, little is known about the role of endothelial sEH in brain ischemia. We generated transgenic mice with endothelial-specific expression of human sEH (Tie2-hsEH), and assessed the effect of transgenic overexpression of endothelial sEH on endothelium-dependent vascular reactivity and ischemic injury following middle cerebral artery occlusion (MCAO). Compared to wild-type, male Tie2-hsEH mice exhibited impaired vasodilation in response to stimulation with 1 µM acetylcholine as assessed by laser-Doppler perfusion monitoring in an in-vivo cranial window preparation. No difference in infarct size was observed between wild-type and Tie2-hsEH male mice. In females, however, Tie2-hsEH mice sustained larger infarcts in striatum, but not cortex, compared to wild-type mice. Sex difference in ischemic injury was maintained in the cortex of Tie2-hsEH mice. In the striatum, expression of Tie2-hsEH resulted in a sex difference, with larger infarct in females than males. These findings demonstrate that transgenic expression of sEH in endothelium results in impaired endothelium-dependent vasodilation in the cerebral circulation, and that females are more susceptible to enhanced ischemic damage as a result of increased endothelial sEH than males, especially in end-arteriolar striatal region.

## Introduction

Epoxyeicosatrienoic acids (EETs) produced in brain play an important role in cerebral blood flow regulation [1]. They are protective against ischemic stroke in vivo and against ischemia-induced cell death in vitro in multiple brain- derived cell types including neurons, astrocytes and endothelial cells [2], [3], [4], [5]. The actions of EETs are predominantly terminated by the enzyme soluble epoxide hydrolase (sEH) which metabolizes EETs to dihydroxyeicosatrienoic acids (DHETs) [6]. sEH is widely expressed in brain including in the cerebral vasculature [4], [7], [8].

Stroke is a major cause of morbidity and mortality worldwide, with lower incidence in premenopausal women compared to men of the same age. However, the risk of stroke in women greatly increases following menopause [9], [10]. This gender difference has therefore been attributed largely to sex hormones, with estrogen in particular affording protection to premenopausal females [11], [12]. Although the mechanism of is not fully understood, and involves many factors and signaling pathways, studies have indicated that sEH is involved in this sexual dimorphism. Soluble epoxide hydrolase is expressed at higher levels in male mice, with higher sEH protein levels in male brains compared to female and also higher EPHX2 transcript levels (gene encoding sEH) in endothelial cells cultured from male brains compared to female [3], [7]. It has been shown that female mice sustain smaller infarct sizes following middle cerebral artery occlusion (MCAO) compared to males; a sex difference which is abolished upon genetic knock-down of sEH. Also, upon ovariectomy of wild-type female mice infarct volumes increase to male levels, however ovariectomized females with genetic ablation of sEH no longer exhibit increased infarct volumes, indicating that ovarian sex hormones contribute to the protection observed in the intact female mice, presumably by decreasing sEH levels [7].

We have shown previously that sEH deletion is protective against ischemic injury in vivo, and also that increased sEH activity is detrimental to neurons in vitro following oxygen-glucose deprivation (OGD) [7], [13]. Much work has focused on the brain as a whole; the function of sEH in the vascular endothelium in vivo has not been investigated. We have demonstrated a role for sEH in vascular responses controlling blood flow in ischemic brain [7], [14]. These studies however, were carried out using sEH knockout mice, where sEH was absent in every cell type; the effect of manipulating sEH solely in the endothelium and the endothelial sEH specifically in ischemic brain has not been studied. In the current study we tested the hypothesis that endothelial sEH plays an important role in regulating cerebral blood flow and thus influences the extent of ischemic injury in the brain following stroke. We also hypothesized that since sEH levels in females are low, forced expression of the human sEH transgene would increase their susceptibility to ischemic insult and abolish the observed sex difference in infarct volumes. To test this we measured endothelium- dependent vasodilation and ischemic injury following MCAO in mice with transgenic expressing of human sEH under the control of the endothelial Tie2 promoter.

## Materials and Methods

### Ethics Statement

The investigation conformed to the Association for Assessment and Accreditation of Laboratory Animal Care AAALAC Accreditation (approved November 2010) and the Office of Laboratory Animal Welfare (LOAW Assurance # A3304-01, approved March 2009). The Oregon Health and Science University Animal Care and Use Committee approved all experiments

### Animals

Transgenic mice expressing Tie2- driven human sEH in endothelial cells on a C57BL/6 genetic background (Tie2-hsEH) have been recently described [15]. Wild type (WT) littermates were used as controls. The study was conducted in accordance with the National Institutes of Health guidelines for the care and use of animals in research, and protocols were approved by the Animal Care and Use Committee of Oregon Health and Science University (Portland, OR, USA).

### Middle Cerebral Artery Occlusion (MCAO)

Mice were subjected to transient focal cerebral ischemia by use of the intraluminal MCAO technique [16]. Briefly, 6-month old WT and Tie2-hsEH mice were anesthetized with isoflurane (1.5–2%) and kept warm using water pads. A transcranial laser-Doppler probe (MBF3D, Moor Instruments, Oxford, UK) was secured on the right temporal side of the head to monitor cerebrocortical perfusion and verify vascular occlusion and reperfusion. A silicone- coated 6–0 nylon monofilament was advanced into the right internal carotid artery via the external carotid artery until laser-Doppler signal dropped to below 30% of baseline. The animal was maintained anesthetized with 1% isoflurane on the surgical station for 60 minutes of occlusion. At the end of occlusion, the filament was withdrawn for reperfusion and the mice were allowed to recover.

### Assessment of Infarct Size

Infarct size was measured 24 hours following MCAO in 2 mm thick coronal sections using 2,3,5-triphenyltetrazolium chloride (TTC) staining. Sections were incubated for 15 minutes in 1.2% TTC in saline at 37°C and fixed in formalin for 24 hours. Sections (5 per animal) were imaged, infarcted (unstained) and viable tissue (stained) areas were measured using MCID software (InterFocus) and integrated across all 5 sections. To discount the influence of edema, infarct size was calculated indirectly by subtracting the uninjured area in the ipsilateral hemisphere from the contralateral hemisphere, then expressing infarct volume as a percentage of the contralateral hemisphere.

### Cranial Window

Anesthesia was induced as above. The head of the mouse was secured in a stereotaxic apparatus with 2% isoflurane in oxygen-enriched air by face mask. The scalp and connective tissue over the parietal cranial bone were removed. Rectal temperature was maintained at 36+/−0.5°C by water pad. An area with 1 mm diameter, 2 mm posterior and 2.5 mm lateral to Bregma, was marked for monitoring by laser-Doppler. A small drill hole was made superior (medial) to the laser-Doppler probe site to expose the dura. The dura was pierced with care not to damage the epidural or pial vessels. A 33-gauge needle connected to a microperfusion pump with PE10 tubing was advanced 1 mm subdurally in a lateral direction (inflow). Another drill hole was made inferior (lateral) to the probe site, and the dura was incised for passive drainage of the superfused fluid (ouflow). Cerebrovascular cortical perfusion was monitored using a laser-Doppler flow probe with a tip diameter of 1 mm (Moor Instruments, Oxford, UK). The probe was placed in a location over the thinned skull that was devoid of large visible blood vessels. The probe position was kept fixed for the entire experiment, and background lighting was minimized and kept constant. For experiments investigating cortical blood flow in response to the endothelium- dependent dilator acetylcholine, subdural superfusion with artificial cerebrospinal fluid (CSF) started 5 minutes after completion of surgery and continued for 20 minutes at a constant rate of 3 µl/min. Following CSF perfusion acetylcholine (1 µM) was superfused for 90 minutes. Artificial CSF and acetylcholine were prepared daily and the solution was pre-warmed to 37°C.

### Immunohistochemistry

Wild-type (WT) and transgenic mouse tissues were prepared and stained as described previously [17]. Briefly, whole brain tissues were fixed in 10% neutral buffered formalin and embedded in paraffin, and serial sections were immunohistochemically labeled with anti-human sEH (1∶4000 dilution; a generous gift from Dr. Bruce Hammock, University of California, Davis, CA, USA). Sections were incubated with donkey anti-rabbit secondary antibody (1∶500 dilution; Vector Laboratories, Burlingame, CA, USA) and visualized using liquid 3,3-diaminobenzidine solution (Dako, Carpinteria, CA, USA). Slides were counterstained with Harris hematoxylin (Harelco, Gibbston, NJ, USA), and image capture using ImageScope software (Aperio Technologies, Vista, CA, USA) was performed as described by the supplier.

### Real-Time Quantitative PCR

Expression of human sEH in mouse brain was confirmed after RNA purification of brain tissue with the RNAeasy Mini kit (Qiagen, Valencia, CA, USA), conversion to cDNA with High Capacity cDNA Archive kit, (Applied Biosystems, Foster City, CA, USA) and real-time PCR using TaqMan probes to murine GAPDH (Mm 99999915_g1) and human EPHX2 (Hs 00157403_m1) (Applied Biosystems).

### LC-MS/MS

Plasma DHET levels were determined by liquid chromatography, tandem mass spectroscopy (LC/MS/MS) as described previously [17]. Perfusates were pooled, spiked with 30 ng PGE2-d4, 10,11- DiHN, and 10,11-EpHep (Cayman) as internal standards, mixed with 0.1 vol of 1% acetic acid in 50% methanol, and extracted by serial passage through Oasis HLB C18 3 ml columns (Waters, Milford, MA, USA). Columns were washed twice with 0.1% acetic acid in 5% methanol, and eluted with methanol into glass tubes containing 6 µl of 30% glycerol in methanol. The methanol was evaporated under a stream of nitrogen gas, and the dried tubes were frozen and stored at −80°C until analysis. Online liquid chromatography of extracted samples was performed with an Agilent 1200 Series capillary HPLC (Agilent Technologies, Santa Clara, CA, USA). Separations were achieved using a Phenomenex Luna C18(2) column (5 µm, 150 x1 mm; Phenomenex, Torrance, CA, USA), which was held at 40°C. Mobile phase A was 85∶15∶0.1 water:acetonitrile:acetic acid. Mobile phase B was 70∶30∶0.1 acetonitrile:methanol:acetic acid. Gradient elution was used; mobile phase percentage B and flow rate were varied as follows: 0% B at 87.5 µl/min at 0 min, ramp from 0 to 2 min to 25% B at 87.5 µl/min, ramp from 2 to 5 min to 50% B at 60 µl/min, ramp from 5 to 23 min to 92.5% B at 60 µl/min, ramp from 23 to 23.5 min to 100% B at 87.5 µl/min, hold from 23.5 to 29 min at 100% B, ramp from 29 to 29.6 min down to 0% B, and hold 0% B to 33 min. Samples were solvated in 100 µl of 40% ethanol. The injection volume was 20 µl. Samples were analyzed in triplicate. Negative ion electrospray ionization tandem mass spectrometry was used for detection. Analyses were performed on an MDS Sciex API 3000 equipped with a TurboIonSpray source (Applied Biosystems). Turbo desolvation gas was heated to 275°C at a flow rate of 5.75 L/min.

### Statistical Analysis

Differences between two groups were evaluated with a *t*-test, while a 2-way ANOVA with post hoc Holm Sidak test was used for comparisons between multiple groups. The criterion for statistical significance was set at *P*<0.05. All values are reported as mean +/−S.E.M.

## Results

**Figure 1:**
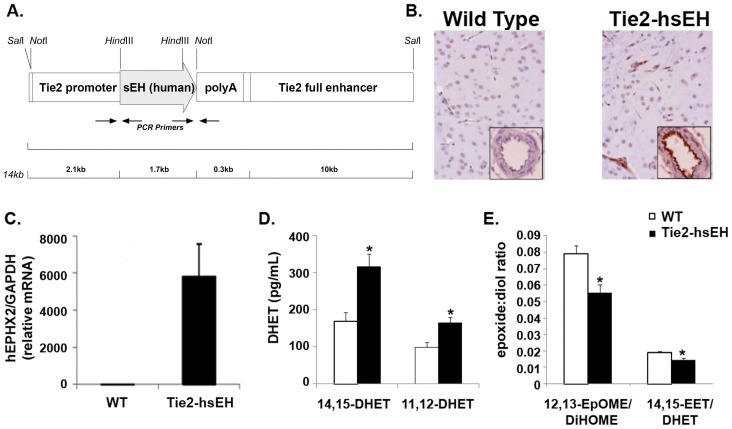
Characterization of Tie2-hsEH mice. A. Schematic of the Tie2-hsEH transgenic construct used to generate mice used in this study. B. Sections of WT (left) and Tie2-hsEH (right) mouse brains immunolabeled for human sEH showing sEH in capillaries and large vessels in Tie2-hsEH. Inserts, blood vessels in cross section, show intense labeling in vascular endothelium in Tie-hsEH. C. Real-time PCR analysis of human sEH levels in brain tissue, showing presence of transgene expression in Tie2-hsEH and none in WT mice. D. LC-MS/MS analysis showing significantly increased 14,15- and 11,12- DHET plasma levels in Tie2-hsEH mice compared to WT (n = 8 WT, n = 13 Tie2-hsEH, *P*<0.05). E. LC-MS analysis of culture medium from WT and Tie2-hsEH aortic endothelial cells showing decreased ratios of 12,13-EpOME:DiHOME and 14,15-EET:DHET released by Tie2-hsEH endothelial cells compared to WT (n = 3 both groups, *P*<0.05).

Mice carrying the Tie2-hsEH transgene expressed human sEH transcript and protein in the brain (Figure 1A and B). Immunolabeling was observed in, and confined to, both large and small vessels, with dense labeling in the vascular endothelium (Figure 1A). Immunolabeling was not observed in any other cell type. These mice displayed increased plasma levels of the EETs hydrolysis products, DHETs, as measured by LC-MS/MS. 14,15-DHETs were increased from 168.68 pg/ml in WT to 317.07 pg/ml in Tie2-hsEH expressing mice and 11,12-DHETs were increased from 99.94 pg/ml to 164.94 pg/ml, respectively (Figure 1D) (n = 8 WT, n = 13 Tie2-hsEH, *P*<0.05). Medium collected from aortic endothelial cell cultures from Tie2-hsEH mice also had lower arachidonic acid and linoleic acid epoxide:diol ratios (14,15-EET:DHET and 12,13-EpOME:DiHOME) as measured by LC-MS/MS, both of which serve as readouts of increased sEH function (Figure 1D) (n = 3, *P*<0.05).

**Figure 2:**
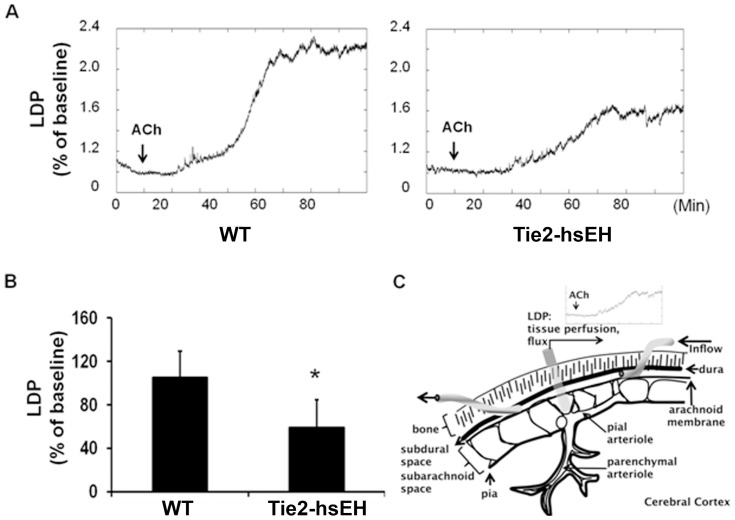
Effect of sEH over-expression on endothelium-dependent cerebral blood flow in male mice. A. Representative traces illustrating reduced LDP response to 1 µM ACh stimulation in Tie2-hsEH compared to WT mice over time. B. Quantification of laser Doppler perfusion (LDP) shows that perfusion is significantly reduced in Tie2-hsEH mice compared to WT. (n = 9 WT, n = 8 Tie2-hsEH, *P*<0.05). C. Schematic representation of cranial window preparation.

Endothelium- dependent vasodilation was impaired in mice carrying the Tie2-hsEH transgene compared to wild-type controls (Figure 2). Cortical blood flow in male mice in response to 1 µM acetylcholine (ACh) was measured by laser-Doppler through a cranial window (Figure 2C). Figure 2A shows representative traces of laser-Doppler perfusion in both genotypes, at baseline and in the 90 minutes following ACh administration. Blood flow was significantly reduced in Tie2-hsEH compared to wild-type mice, with 58.9% (+/−24.3%) increase in blood flow from baseline on stimulation with ACh compared to 105.3% (+/−26%) respectively (Figure 2B) (n = 9 WT, n = 8 Tie2-hsEH, *P*<0.05).

**Figure 3:**
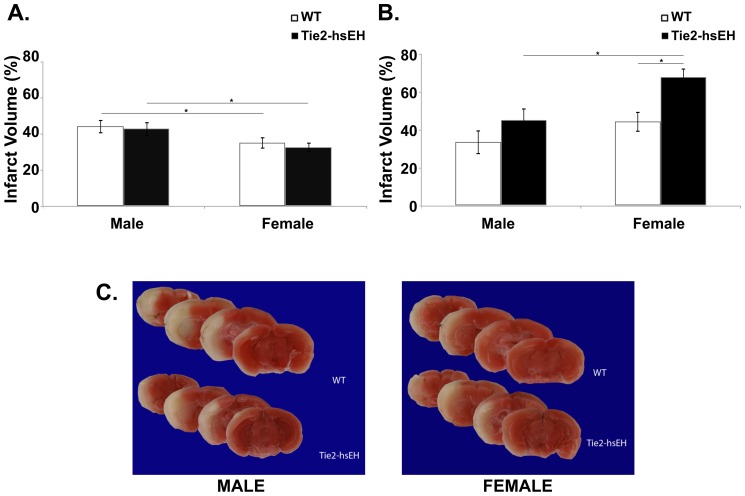
Effect of sEH over-expression on infarct size after MCAO in male and female mice. A. In the cortex, there is no significant difference in infarct volume following MCAO in WT compared to Tie2-hsEH mice in either male or female mice (n = 5 male WT, n = 5 male Tie-hsEH, n = 7 female WT, n = 10 female Tie2-hsEH, *P*>0.05). Infarct volume is reduced in females compared to males in both WT and Tie2-hsEH expressing mice (*P*<0.05). B. In the caudate patamen (CP) there is no significant difference in infarct volume following MCAO in WT compared to Tie2-hsEH male mice, however in females infarct volume is increased in Tie2-hsEH compared to WT control mice (*P*<0.05). The Tie2-hsEH female mice also have an increased infarct volume compared to male Tie2-hsEH mice (*P*<0.05). C.Representative TCC- stained brain slices from WT and Tie2-hsEH mice from male (left) and female (right) mice.

Since increased endothelial expression of sEH impaired ACh- stimulated vasodilation at baseline, we next investigated whether this endothelial dysfunction is deleterious following MCAO in Tie2-hsEH compared to WT mice. Male mice were subjected to 60 minutes of MCAO and infarct volume was assessed following 24 hours of reperfusion. Infarct volume was assessed histologically by TTC labeling (Figure 3C). In male mice, no difference in infarct size was observed in either cortex (Figure 3A), caudate putamen (striatum; Figure 3B) or the whole hemisphere (not shown) between Tie2-hsEH and WT mice (Figure 3B) (n = 5, *P*>0.05).

**Figure 4:**
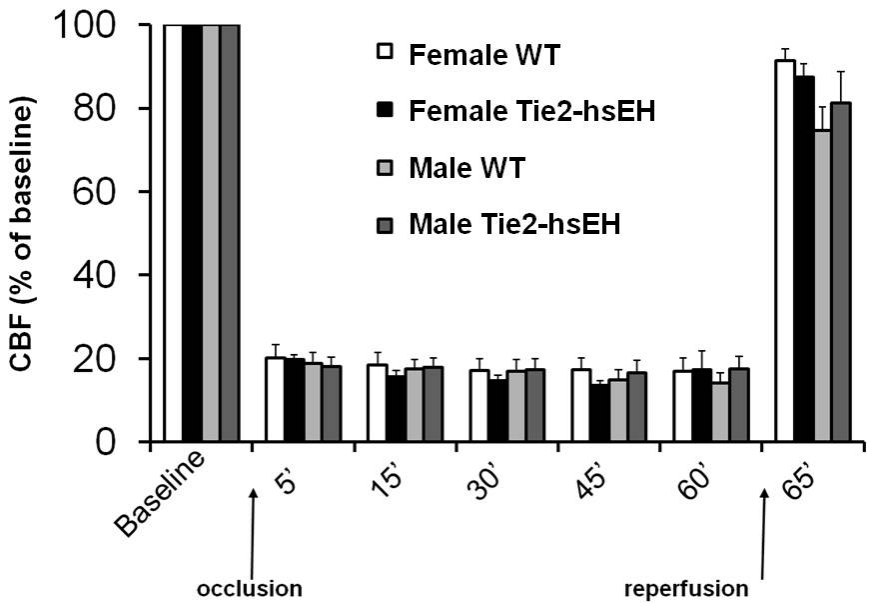
Cerebral blood flow (CBF) during MCAO. No significant differences in blood flow changes are observed between wild type and Tie2-hsEH mice either during MCAO or immediately following MCAO.

Sex differences have been reported in sEH expression; females express lower levels of sEH in the brain affording them protection from ischemic insult [7]. We therefore hypothesized that increasing levels of sEH in female endothelium would render them more susceptible to ischemic damage. Female mice were subjected to 60 minutes of MCAO, followed by 24 hours of reperfusion. Brains were analyzed by TTC labeling, Figure 3C shows representative images of sections from WT and Tie2-hsEH mouse brains depicting smaller infarct area (white) in WT compared to Tie2-hsEH brain. On quantification, infarct volume was significantly larger in the caudate putamen (striatum) of Tie2-hsEH mice compared to their WT counterparts (67.78+/−4.52% compared to 44.34+/−5.40% respectively; n = 7 WT, n = 10 Tie2-hsEH, *P*<0.05, Figure 3B). There was however no difference in the cortex (Figure 3A) or whole hemisphere (not shown) between the Tie2-hsEH and WT mice. There was however a sex difference in infarct volume in the WT cortex (Figure 3A), in which females sustained smaller infarcts compared to males (35.23+/−3.26% compared to 44.34+/−3.85% respectively; n = 7 female, n = 5 male, *P*<0.05); this difference persisted in the Tie2-hsEH mice (32.77+/−2.78% female, 43.12+/−3.85% male; n = 10 female, n = 5 male, *P*<0.05). In the striatum, while there was no significant sex difference in infarct size in the WT mice, a sex difference was revealed in mice over-expressing hsEH, with females exhibiting larger infarct volumes than males (67.78+/−4.52% compared to 45.14+/−6.39% respectively; n = 10 female, n = 5 male, *P*<0.05). To ensure that the insult sustained was comparable between groups and that this observed difference is not due to varying levels of occlusion, cerebral blood flow was measured during and immediately after MCAO (Figure 4); no difference in blood flow was observed during the MCAO procedure.

## Discussion

We report the following new findings: (1) Endothelial over-expression of sEH impairs ACh- induced increase in cerebral blood flow in males. (2) Endothelial over-expression of sEH does not exacerbate ischemic damage following MCAO in males, however (3) females are susceptible to increased ischemic damage following MCAO due to increased sEH expression in brain endothelium, especially in end-arteriolar striatal region. (4) In the cortex, endothelial over-expression of sEH does not impact the observed sex difference in infarct size following MCAO, however (5) in the striatum while there is no sex difference in wild type mice, endothelial over-expression of sEH renders females more susceptible to ischemic damage than males.

We have previously demonstrated a role for sEH in mediating vascular function. EETs are known vasodilators; it would follow that knocking out sEH, which hydrolyzes EETs would increase their concentration. Mice with global deletion of the sEH gene display higher cerebral blood flow levels during MCAO compared to WT controls [14]. The current study demonstrates the reverse effect on cerebral blood flow regulation; male mice expressing higher levels of endothelial sEH display an impaired vasodilator response to stimulation with ACh. In addition to causing the release of nitric oxide (NO), ACh is known to release endothelial EETs in the cardiac circulation [18]. Thus, ACh stimulation causes release of EETs, resulting in vasodilation; with increased sEH present in our mouse model, this should increase the hydrolysis product of EETs, which is indeed what we found. These mice have higher circulating blood levels of 14,15- and 11,12- DHET as well as a lower ratio of 14,15- EET:DHET released by endothelial cells, confirming recent studies which have also shown reduced epoxide:diol ratios and decreased 14,15-EET in plasma and endothelial cells of other tissues from mice expressing this human sEH transgene [15], [19]. This therefore presumably leads to lower levels of bioavailable EETs, and thus a reduced vasodilator response to ACh.

Endothelial response to acetylcholine has been used as a benchmark for measuring endothelial dysfunction [20], [21]. It has been suggested that sEH contributes to endothelial dysfunction; indeed inhibition of sEH ameliorates endothelial dysfunction in mouse models of diabetes and hypertension [22]. This current study adds further substance to this notion; we show that increasing sEH levels in endothelial cells leads to impaired ACh- vascular responses in male mice, and thus endothelial dysfunction.

Male mice expressing the Tie2-hsEH transgene did not exhibit larger infarct sizes following MCAO. Although perhaps surprising, this can be explained by the fact that sEH is already highly expressed in males, and therefore higher levels may not be deleterious in terms of ischemic injury [7]. Interestingly, over-expression of sEH in the endothelium also had no adverse effects on infarct size in the heart [15].

As discussed earlier, sEH expression in the brain is sexually dimorphic. In whole brain extracts, males exhibit higher protein levels than females; this is reflected in higher 14,15-DHET levels in males than females [7]. In vitro, we have also shown that male endothelial cells are more susceptible to death following oxygen-glucose deprivation than females; a phenomenon attributable to higher expression of sEH in males than females, resulting in higher EETs levels in females than males [3]. In this study, possession of the Tie2-hsEH transgene altered response to injury in females, but not males. Increased sEH expression had a deleterious effect on infarct size following MCAO in females only in the caudate putamen (striatum), but not in the cerebral cortex. This is consistent with the idea that striatum is an end-arteriolar region devoid of collaterals, unlike the cerebral cortex which is rich in collateral vascular networks, and is therefore less dependent on vascular mechanisms. In terms of direct comparison of the sexual dimorphic response to MCAO, our data supports previous findings that females sustain smaller cortical infarcts than males [7]. Expression of the human sEH transgene in the endothelium did not alter this sex difference. In the striatum however, while there was no difference between male and female infarct volume in the wild-type mice, a sex difference was revealed in the mice carrying the hsEH transgene, with females actually exhibiting larger infarcts than males. A possible explanation for this is that, as mentioned, the striatum is an end-arteriolar region lacking collateral supply. It has been suggested that EETs are a component of the endothelium- derived hyperpolarizing factor (EDHF) response in small caliber vessels in the brain but not of large vessels [23], [24], [25], [26]. Therefore, increasing the expression of sEH in females, which have lower endogenous expression than male, in an area of the brain dependent on blood supply from small caliber vessels will have a more profound effect than in the males or than in the cortex.

It is important to note that this study explores the effect of genetic manipulation of sEH specifically in the endothelium, however the effects are still within the context of the whole brain. Other cell types within the brain, including perivascular astrocytes and neurons have been shown to produce both EETs and sEH. In the human brain, sEH is expressed throughout the brain in neurons, oligodendrocytes, astrocytes, endothelial cells and also smooth muscle cells of arterioles [8]. Studies have shown that astrocyte- derived EETs are involved in neurovascular coupling and vasodilation [4], [27], and perivascular nerve- derived EETs mediate neurogenic vasodilation of the cerebral vasculature [28]. Therefore although in the current study we are investigating the effects of increasing sEH expression in endothelial cells specifically, the results observed here may be dampened, or compensated for, by vasodilation induced by EETs generated by other cell types within the brain, as it is likely that EETs derived from multiple sources act in concert to exert their protective role.

In summary, the current study demonstrates a role for endothelial sEH in the regulation of cerebral blood flow. We also confirm the sexual dimorphism observed in endothelial sEH function, with increased sEH expression being detrimental to females but not males in terms of infarct size following MCAO. These findings suggest that endothelial sEH is a key regulator of cerebrovascular endothelial function and an important contributor to the sexually dimorphic response to cerebral ischemic injury.
